# Motivational Determinants of Recreational Padel Participation: A Comparative Analysis Across Age and Gender

**DOI:** 10.3390/sports13110377

**Published:** 2025-11-03

**Authors:** Zlatan Bilić, Petar Barbaros, Vedran Dukarić, Tea Lovreković, Lidija Petrinović

**Affiliations:** Faculty of Kinesiology, University of Zagreb, 10000 Zagreb, Croatia; petar.barbaros@kif.unizg.hr (P.B.); vedran.dukaric@kif.unizg.hr (V.D.); tea.lovrekovic@student.kif.unizg.hr (T.L.); lidija.petrinovic@kif.unizg.hr (L.P.)

**Keywords:** motivation, recreational sport, padel, questionnaire, age difference, gender differences

## Abstract

The aim of this study is to examine the differences in motivation when playing recreational padel regarding gender and age. The sample included 201 respondents (M = 131, F = 70) with an average age of 31 ± 7.07 years. Data was collected online via the Sport Motivation Scale-II (SMS-II) questionnaire. Spearman correlation presented the highest correlation between intrinsic and introjected (r = 0.658) motivation forms. Furthermore, results were analyzed using the Mann–Whitney U test. The results showed a statistically significant difference in motivation between men and women exclusively in the dimension of introjected regulation (M = 107.54, F = 88.76; *p* = 0.03), while no difference was found in the other dimensions. Also, introjected regulation was found to have the highest impact on differences across age (18–30 yr = 94.39; 31–50 yr = 110.59; *p* = 0.05). The research shows that padel as a recreational activity has a similar motivational effect on different age and gender groups, apart from introjected regulation, where men show higher results. Overall, the motivation to engage in padel largely depends on personal interests, enjoyment and perceived benefits, while gender and age have a limited influence, which coincides with some of the previous literature. Since research on motivation in the context of padel is still rare, this research contributes to the understanding of the factors that influence recreational engagement in this sport.

## 1. Introduction

Padel is typically played in pairs on a compact 20 × 10 m court with synthetic turf, bounded by glass and metallic mesh walls that permit the ball to rebound, giving the game its distinctive continuous and dynamic character [1]. Also referred to as padel tennis, it is a relatively new racket sport characterized by dynamic movement patterns and the integration of elements from several other sports. The possibility of the ball rebounding off the back and side walls affects the dynamics of the game, increasing the tempo and frequency of strokes, while the physical load on the players remains at a moderate level [1]. To begin learning padel, a high level of technical skill is not required, and it is accessible to people of all age groups. Literature [2] states that beginners, with one group and one individual lesson per week, need approximately two months to acquire the basic elements of the game. To progress to an intermediate level of play, where more varied strokes and deeper tactical understanding are developed, the estimated duration of the process is four to six months. In Padel, it is easier to find an appropriate balance between four players, which ensures a higher level of fun and excitement, while longer rallies increase players’ enjoyment during the game [3]. One of the reasons for the growing popularity of padel compared to other racket sports is its accessibility where beginners do not need advanced technical skills, the game can be practiced both indoors and outdoors, and the necessary equipment is relatively affordable [4,5,6]. Due to its distinctive characteristics, padel has recently become a more frequent focus of scientific interest than many other racket sports [5,7,8,9]. Therefore, recreational padel has become an increasingly popular way of maintaining fitness and an active lifestyle among all age categories, especially adults. Since it is widely played in a recreational environment, this sport has the potential to contribute to promoting physical activity and healthy habits among young people and adults [6].

It is well known that regular sport and physical activity bring numerous benefits, not only physically but also mentally, as they improve health and quality of life and make people more satisfied [10]. Despite this, the number of physically inactive people is growing, especially in adulthood, and inactivity and a sedentary lifestyle are leading to health risks in modern society. The results of various studies show that over 60% of the population is physically inactive [11]. Physically active people have a lower risk of cardiovascular diseases, certain cancers, obesity, depression, and high blood pressure [12]. Motivation for initiating sport activity plays a crucial role in an individual’s overall health and well-being. Motivation explains why a person behaves in a certain way at a certain moment, i.e., what drives them internally and externally toward achieving a goal and keeps them engaged in a certain activity [13].

Since motivation includes the desire for success and progress, its role in sport is extremely important. In the sports context, motivation is a process that directs the behavior of athletes and determines its intensity and persistence, and its goal is to encourage self-confidence, continuous work and improvement of sports skills. In recent years, one of the most frequently applied and studied theoretical foundations in the study of motivation (in sport) is the Self-Determination Theory (SDT) [14]. SDT is a complex macro theory that views human motivation from the perspective of volition and autonomy and states that a higher level of autonomous motivation contributes to success and psychological well-being.

Self-determination implies that a person makes decisions and initiates their behavior freely and internally, without the influence of external factors. This concept is viewed as a continuum between intrinsic and external motivation. The level of self-determination increases as a person moves away from external influences and toward activities that are aligned with their personal values. Athletes and recreational participants often have multiple motives simultaneously for engaging in an activity. According to SDT [15], the key elements that encourage self-determined behavior are the satisfaction of three basic psychological needs. SDT identifies six types of motivation regulations that differ in the level of autonomy and control. These forms of regulation are presented in a continuum from the least to the most autonomous, whereby greater autonomy in motivation leads to more positive outcomes. In conclusion, this theory provides a comprehensive framework for studying external and intrinsic regulation and their impact on participation in sports activities. Different motives may have different effects on athletes’ engagement and the benefits that result from participating in sport. Research has shown that intrinsic motivation and integrated regulation, which stem from personal interest, more often lead to long-term commitment and fulfillment in sport, while external forms of motivation may have a less stable impact on participation in sport [16].

As participation in sport continues to increase, it is important to identify the factors that motivate individuals to engage in this physical activity. Therefore, the aim of this paper is to investigate the motives that encourage people of different genders and age groups to engage in recreational padel and to contribute to the development of effective strategies that will encourage a greater number of people to take up this growing sport. Based on previously conducted research on similar topics, a potential influence of sociodemographic factors on individuals’ motivational patterns was observed. Men and women may be motivated by different aspects of recreational sport, such as competition, social interaction, or personal enjoyment. In addition, motivation can vary across age, with younger adults often seeking progress and excitement, while older adults may focus more on health or social factors. Since evidence on padel is scarce, these expectations are mainly exploratory. Therefore, the authors hypothesize that there will be statistically significant differences in motivation for participating in recreational padel according to age and gender.

## 2. Materials and Methods

### 2.1. Experimental Approach

Data was collected between 11 January 2025, and 24 March 2025. The questionnaire was sent to participants electronically, meaning that data was collected online using the Google Forms platform (accessed on 5 April 2025). The criteria for participation in the study and completion of the questionnaire included men and women aged between 18 and 50 who play recreational padel at padel centers in Croatia. The level of experience in padel was not a limiting factor for inclusion in the study. Participation in the study was entirely voluntary. Participants were informed that the questionnaire was anonymous and that the collected data would be used solely for the purposes of this research. The questions were closed-ended, and participants could stop completing the questionnaire at any time if they felt discomfort or stress when answering questions, as some items related to personal motives, self-assessment, or social pressures. The average time to complete the questionnaire was 4 to 5 min.

### 2.2. Sample of Participants

The sample consisted of recreational male and female padel players in the Republic of Croatia aged between 18 and 50 years. The participants were divided into groups according to gender (male and female) and age (younger adults—between 18 and 30 years and older adults—between 31 and 50 years). A total of 201 respondents participated in this study. A power analysis was conducted in G*Power 3.1.9.7 [17] in order to determine the minimum sample size required to test differences between two groups using the Mann–Whitney U test. The following parameters were used in the calculation: medium effect size (Cohen’s d = 0.5), significance level α = 0.05, and statistical power 1 − β = 0.80. The results indicated that, to achieve adequate power, a minimum of 118 participants (i.e., 59 per group) was required. The total number of participants in this study (N = 201) corresponds with the previously conducted power analysis, which ensured adequate power for testing the stated hypotheses.

Regarding the gender structure of the respondents, the sample included 65% male padel players (n = 131) with an average age of 30.92 ± 6.74 years and 35% female padel players (n = 70) with an average age of 30.83 ± 7.70 years.

Regarding the age structure of the respondents, participants were between 18 and 50 years old, with a mean age of 30.89 ± 7.07 years. Based on two theoretically defined age groups, the sample included 59% (n = 119) younger adults and 41% (n = 82) older adults. The most common age at the time of the study was 24 years (n = 19), while 50% of the respondents were 29 years or younger, and the other 50% were 29 years or older. The study was conducted in accordance with the Declaration of Helsinki and approved by the Ethics Committee of the Faculty of Kinesiology, University of Zagreb (Approval No. 02/2025).

### 2.3. Sample of Variables

The level and type of motivation for recreational participation in padel were measured using the SMS-II scale (Sport Motivation Scale) [17]. The Croatian version of the instrument was applied, which has previously undergone psychometric evaluation and demonstrated satisfactory reliability and validity for use among the Croatian population [18]. The SMS-II assesses different forms of motivation in a sports context, classified along the self-determination continuum, from fully autonomous (intrinsic, integrated, and identified) to controlled (introjected and external), as well as amotivation (lack of motivation). To compare different types of motivational regulation, unique indicators were constructed for each form of regulation. Specifically, simple additive indices were created for intrinsic, integrated, identified, introjected, external regulation, and amotivation by summing the values of all items corresponding to each type of regulation for each participant. To make the constructed indices comparable, the sum of item scores was divided by the number of items for each type of regulation within each subscale. As a result, the range of values for all constructed indices is from 1 to 7. On all indices, lower scores indicate a lower presence of a particular form of motivation for recreational padel, while higher scores indicate a higher level of motivation.

The questionnaire consists of two parts. The first part collects basic sociodemographic data of the respondents, including gender and age. The second part includes 18 statements accompanied by a seven-point Likert-type scale (from 1—“Does not correspond at all” to 7—“Corresponds completely”).

### 2.4. Data Analysis

The analysis and processing of the collected data were performed using the statistical software package for social sciences SPSS 21 (IBM Corp., Armonk, NY, USA). For all observed variables, basic descriptive statistical parameters were calculated. Normality of distribution was determined with the Shapiro–Wilk test. The presented data does not have normally distributed results. The Mann–Whitney U test was conducted to evaluate the hypotheses and to determine differences between genders and age groups in the observed variables. To test the relationships among the observed variables, Spearman’s rank correlation coefficient was applied. All statistical tests were conducted at a significance level of 5% (*p* = 0.05). Also, effect size (η2) calculations (partial eta squared; small (η2 ≥ 0.01), medium (η2 ≥ 0.06), and large (η2 ≥ 0.14)) were used to estimate the magnitude of the result.

## 3. Results

According to the degree of agreement with individual items of the instrument measuring the level and type of motivation for recreational padel, participants most strongly agreed with the statement that they play padel because it makes them feel better about themselves when they do it (M ± SD = 6.31 ± 0.98). Conversely, they agreed with the statement that they are no longer sure and feel that their place is not in sports (M ± SD = 1.35 ± 0.97). Looking at the mean values of the six constructed indices, participants agreed most strongly with items measuring intrinsic motivation (M ± SD = 6.04 ± 1.09), less with integrated, identified, and introjected regulation, and least with items measuring external regulation (M ± SD = 1.98 ± 1.16) and amotivation (M ± SD = 1.67 ± 0.92). Selected descriptive statistics of the constructed indices are shown in Table 1.

To analyze the interrelationship among different forms of motivational regulation, Spearman correlation coefficients were calculated. The results are presented in Table 2.

Reviewing the results in Table 2, a strong positive correlation is visible among autonomous forms of regulation. Intrinsic regulation shows statistically significant positive correlations with integrated regulation (*p* = 0.383; *p* < 0.001), identified regulation (*p* = 0.658; *p* < 0.001), and introjected regulation (*p* = 0.307; *p* < 0.001). This indicates that individuals who engage in recreational padel for internal reasons often simultaneously experience high levels of identification and internalization of goals, reflecting stable and high-quality motivation. Conversely, intrinsic motivation is negatively correlated with amotivation (*p* = *−*0.214; *p* < 0.01), showing that participants who find padel personally meaningful and enjoyable are less likely to feel aimless or unmotivated. Integrated regulation shows moderate positive correlations with identified regulation (*p* = 0.424; *p* < 0.001) and introjected regulation (*p* = 0.397; *p* < 0.001), while negatively correlating with amotivation (*p* = −0.311; *p* < 0.001). Individuals who see padel as part of their personal values also report lower levels of demotivation. Identified regulation is positively associated with introjected regulation (*p* = 0.377; *p* < 0.01) and, to a weaker but significant extent, with external regulation (*p* = 0.186; *p* < 0.001), and negatively with amotivation (*p* = *−*0.186; *p* < 0.05). While some participants view playing padel as a means of personal goal fulfillment, external motivation factors are present for some. Introjected regulation shows a positive correlation with extrinsic regulation (*p* = 0.290; *p* < 0.001), indicating that for some participants, motivational processes are linked to feelings of obligation or the need to meet others’ expectations. External regulation positively correlates with amotivation (*p* = 0.254; *p* < 0.001), suggesting that participants primarily motivated externally may gradually show signs of losing internal motivation. In conclusion, the Spearman correlation analysis supports the theoretical SDT model, showing that similar forms of regulation are positively related, while opposing forms correlate negatively. More autonomous forms of motivation, such as intrinsic, integrated, and identified motivation, are positively interrelated and play a key role in maintaining high levels of engagement in recreational padel. Conversely, a higher reliance on external regulation and external sources of motivation may increase the risk of amotivation, highlighting the importance of fostering internal motivation for long-term participation in sport.

According to the results of the conducted test (Table 3), it was determined that men and women do not differ statistically significantly in intrinsic motivation (Mann–Whitney U test = 4342.500; *p* = 0.529; *p* > 0.05), integrated regulation (Mann–Whitney U test = 3903.000; *p* = 0.080; *p* > 0.05), identified regulation (Mann–Whitney U test = 3861.500; *p* = 0.064; *p* > 0.05), external regulation (Mann–Whitney U test = 4510.000; *p* = 0.845; *p* > 0.05), and amotivation (Mann–Whitney U test = 4499.500; *p* = 0.820; *p* > 0.05). On the other hand, it was found that men and women do differ statistically significantly in the dimension of introjected regulation (Mann–Whitney U test = 3728.500; *p* = 0.029; *p* < 0.05). Based on the obtained results, the first hypothesis, stating that there is a statistically significant difference in motivation for engaging in recreational padel between men and women, is not supported, since no statistically significant differences were found between men and women in any variable except for the introjected regulation dimension. The results presented in Table 3 indicate that younger and older adults did not differ significantly in any of the observed variables. Consequently, the second hypothesis, stating that there is a statistically significant difference in motivation for engaging in recreational padel between different age groups, is also not supported.

## 4. Discussion

The main objective of the present research was to explore motivational differences for recreational padel participation across age and gender groups. The first hypothesis (H1: There is a statistically significant difference in motivation for engaging in recreational padel between men and women) posited the expectation that there would be a statistically significant difference in overall motivation between men and women. The research results, obtained using the Mann–Whitney U test, showed that a statistically significant difference exists only in one dimension—introjected regulation—while no statistically significant differences were found in the other dimensions. Therefore, the first hypothesis is rejected. Regarding the introjected regulation dimension, which refers to internal pressure to behave in a certain way and feelings of shame or guilt if one does not engage in a particular activity, the results were higher among men compared to women. One possible explanation, although speculative, is that men may feel a social or personal obligation to participate in recreational sports such as padel in order to meet societal expectations or maintain a particular self-image. Additionally, there is a larger number of men currently participating in recreational padel in Croatia, which may lead men to feel a stronger need to demonstrate their abilities or satisfy social expectations related to masculinity and physical fitness.

These results can be compared with the findings of Frederick and Ryan [19], who examined gender differences in motivation for physical activity in 376 participants. Their study also showed that men more frequently exhibit introjected motivation, particularly in the context of achieving personal goals, experiencing a sense of accomplishment, and developing identity through sport, as men often link sport with personal value and social status.

This finding partially contrasts with the results [4] that reported higher motivation related to competition and social aspects among women over 40 years of age, as well as health-related motives in players with 6 months to 3 years of padel experience. While their study highlighted gender- and age-specific differences, particularly favoring women in social and competitive motives, the results present a more uniform motivational pattern across both genders, with the exception of introjected regulation. One possible explanation is that the Croatian recreational padel population is relatively younger (M = 31 years), which may attenuate some of the gender- and age-related differences observed in older Spanish samples [4]. On the other hand, the absence of differences in the other motivational dimensions (intrinsic motivation, identified regulation, integrated regulation, external regulation, and amotivation) suggests that padel appears to attract individuals of both genders for similar, more autonomous and positive reasons, which is common in social recreational sports.

The second hypothesis (H2: There is a statistically significant difference in motivation for engaging in recreational padel between different age groups) also suggested the existence of differences in motivational dimensions between two age groups: younger adults (18–30 years) and older adults (31–50 years). The results obtained using the Mann–Whitney U test showed that there are no statistically significant differences in any of the observed motivational dimensions, and thus the second hypothesis is also rejected. In the introjected regulation dimension, the *p*-value was 0.051, which does not reach the conventional level of statistical significance. This non-significant trend may nevertheless be of interest for future research. In a larger or differently structured sample, or with a significance threshold above 5%, this could potentially reach statistical significance. In this case, older adults show higher levels of introjected regulation compared to younger adults, which could be explained by their greater tendency to feel obligated to participate in padel to meet personal or socially conditioned standards. Similar results have been observed in other recreational sports [20], where older age groups demonstrated greater motivation based on duties and social norms. However, in this study, the difference does not exceed the significance threshold and cannot be considered statistically significant, though it represents an interesting avenue for future research.

The absence of differences in age-related motivation is partly in line with the study [21] where adult women who played padel reported better mood states compared to inactive women, although their quality of life scores (EuroQol-5D) were not substantially different, except for higher values on the visual analog scale. Their findings emphasize that padel positively affects psychological well-being, especially among women, whereas our study highlights that such benefits appear to be independent of age. Taken together with the present findings, this suggests that padel may serve as a universal motivator across different demographic categories, with psychological and social benefits that transcend age or gender boundaries [21].

The absence of differences in all motivational dimensions between younger and older adults indicates that recreational padel players, regardless of age, are motivated by similar internal and external factors. Since recreational padel does not require high levels of fitness, is learned quickly, played in pairs, and has a strong social component, the motivational factors attracting younger participants are often the same as those attracting older participants—such as social interaction, fun, and a healthy lifestyle. Other research has also shown that gender and age do not necessarily represent key factors in motivation for recreational sports. For example, among Norwegian adolescents, it was found that, although girls had slightly higher intrinsic motives, gender did not significantly affect physical activity participation [22]. Authors [23] analyzed differences in exercise motivation among students across various sociodemographic categories and found no significant gender differences in motivation. Similarly, a study [24] found no significant differences between younger and older athletes in soccer, handball, and water polo, suggesting that motivational patterns can be similar regardless of age. Findings of segmenting recreational tennis players by their level of engagement reported no statistically significant differences in demographic variables. In conclusion, the lack of differences indicates the universal appeal of padel as a form of physical activity [25]. Padel, as a relatively new, dynamic, and socially attractive sport, appeals to individuals of different age groups and genders for similar reasons. Future research could more closely investigate the role of social factors, sports habits, and individual goals in shaping motivation for recreational padel participation. Looking at individual motivational dimensions, the results show that participants mostly agreed with items related to intrinsic motivation, particularly with the statement that they play padel because it makes them feel better about themselves. The lowest agreement was with items related to amotivation, where only 3% of participants agreed with the statement “I am unsure; I feel I don’t belong in sport.” The high level of intrinsic motivation for recreational padel can be explained by the nature of the sport itself, as it is dynamic, fun, accessible, and sociable, which enhances feelings of social connectedness and enjoyment.

Participants engage in recreational padel likely because the activity provides personal satisfaction and positive emotional experiences. Conversely, the lowest agreement with the amotivation dimension indicates that participants rarely experience feelings of coercion, meaninglessness, or lack of purpose in relation to padel participation. The absence of amotivation shows that most participants have personal motivation and a clear purpose for engaging in this activity. This result is logical, given that participation in the study was voluntary and participants actively play padel, so it is reasonable that most do not question the purpose of their involvement. These findings indicate that participants perceive recreational padel as an activity that provides enjoyment and personal satisfaction and that they rarely experience a lack of motivation. This aligns with Self-Determination Theory (SDT) [15], which posits that intrinsic motivation is the most favorable form of motivation, leading to long-term participation, greater commitment, and higher satisfaction, emphasizing the importance of satisfying basic psychological needs for autonomy, competence, and relatedness. In relation to presented motivation factors, winning the point or a padel match highly affects increasing motivation for participating in this sport [26]. Furthermore, high demands and physiological stress in a constrained area also significantly affect increased motivation [27].

It is also necessary to highlight certain limitations of this study. First, the sample of participants was unevenly distributed by age and gender. Specifically, the majority were men (65%) and younger adults (59%), which may affect the generalizability of the results and complicate the assessment of actual differences among age and gender groups. Another limitation of this study is that specific characteristics related to sports practice, such as the total number of weekly training sessions and years of experience in playing padel, were not considered. Furthermore, the data were collected via an online survey, which presents the possibility of inaccurate or insincere responses, distractions, or difficulties, and excludes individuals without access to digital platforms. Since the questionnaire is based on self-assessment, there is room for subjectivity in responses and susceptibility to participant bias (e.g., presenting themselves in a more favorable light or misjudging their own motives). Also, because of the cross-sectional and self-report design, causal conclusions cannot be drawn, and the testing of multiple motivational dimensions without adjustment increases the risk of Type I error; therefore, the findings should be interpreted with caution.

## 5. Conclusions

The obtained results showed that sex and age differences do not manifest across all motivational dimensions, leading to the rejection of both hypotheses. Specifically, a statistically significant difference between men and women was found only in the dimension of introjected regulation, where men scored higher. No differences were found in any other dimensions, indicating that men and women share similar reasons for participating in this sport. On the other hand, no statistically significant differences were observed in any dimension between younger adults (18–30 years) and older adults (31–50 years). However, it is worth noting that in the dimension of introjected regulation, a marginal significance value was found (*p* = 0.051), which may indicate a potential difference in a larger sample.

Overall, the results of this study suggest that padel motivates different groups of people in a similar way, regardless of age or sex. Its recreational nature, accessibility, and social character make it suitable and appealing to a broad population, which is an important finding in the context of promoting physical activity and a healthy lifestyle. Since motivation to engage in sports and recreational activities is considered a key predictor of long-term participation, the obtained results provide valuable insights for understanding the needs and motives of different groups of recreational participants. These insights can contribute to developing targeted strategies to engage and encourage more people to participate in this sport.

## Figures and Tables

**Table 1:** Selected descriptive parameters of the constructed indices.

	Mean	SD	−95% CI	+95% CI
Intrinsic	6.04	1.09	0.99	1.21
Integrated	5.75	1.28	1.16	1.42
Identified	5.27	1.33	1.21	1.47
Introjected	4.30	1.17	1.06	1.29
External	1.98	1.16	1.05	1.28
Amotivation	1.67	0.92	0.84	1.02

Legend: Mean—mean value; SD—standard deviation; −95% CI, 95% CI—confidence intervals.

**Table 2:** Spearman correlation coefficients between analyzed conceptual variables.

	Intrinsic	Integrated	Identified	Introjected	External	Amotivation
Intrinsic	1	0.383 ***	0.658 ***	0.307 ***	0.069	−0.214 **
Integrated		1	0.424 ***	0.397 ***	0.043	−0.311 ***
Identified			1	0.377 ***	0.186 **	−0.168 *
Introjected				1	0.290 ***	0.030
External					1	0.254 ***
Amotivation						1

Legend: *** *p* < 0.001; ** *p* < 0.01; * *p* < 0.05.

**Table 3:** Age and gender related differences (Mann–Whitney U test) in the observed variables.

	Variable	Mean Rank (M)	Mean Rank (F)	U	*p*	η^2^		Variable	Mean Rank (18–30)	Mean Rank (31–50)	U	*p*	η^2^
Gender	Intrinsic	99.15	104.46	4342.50	0.53	0.00	Age	Intrinsic	104.05	96.58	4516.50	0.36	0.00
	Integrated	106.21	91.26	3903.00	0.08	0.02		Integrated	106.55	92.95	4219.00	0.10	0.02
	Identified	95.48	111.34	3861.50	0.06	0.08		Identified	100.15	102.24	4777.50	0.80	0.00
	Introjected	107.54 *	88.76 *	3728.50 *	0.03 *	0.02 *		Introjected	94.39	110.59	4092.50	0.05	0.02
	External	100.43	102.07	4510.00	0.85	0.00		External	102.36	99.02	4717.50	0.68	0.00
	Amotivation	100.35	102.22	4499.50	0.82	0.00		Amotivation	97.12	106.63	4417.00	0.23	0.00

Legend: * marked values are significant when *p* < 0.05.

## Data Availability

The data presented in this study are available on request from the corresponding author. The data are not publicly available due to privacy concerns.
